# Molecular Hallmarks of Adult T Cell Leukemia

**DOI:** 10.3389/fmicb.2012.00334

**Published:** 2012-09-17

**Authors:** Makoto Yamagishi, Toshiki Watanabe

**Affiliations:** ^1^Laboratory of Tumor Cell Biology, Department of Medical Genome Sciences, Graduate School of Frontier Sciences, The University of TokyoMinato-ku, Tokyo, Japan

**Keywords:** HTLV-1, ATL, genome, epigenetics, miRNA, signal transduction

## Abstract

The molecular hallmarks of adult T cell leukemia (ATL) comprise outstanding deregulations of signaling pathways that control the cell cycle, resistance to apoptosis, and proliferation of leukemic cells, all of which have been identified by early excellent studies. Nevertheless, we are now confronted the therapeutic difficulties of ATL that is a most aggressive T cell leukemia/lymphoma. Using next-generation strategies, emerging molecular characteristics such as specific surface markers and an additional catalog of signals affecting the fate of leukemic cells have been added to the molecular hallmarks that constitute an organizing principle for rationalizing the complexities of ATL. Although human T cell leukemia virus type 1 is undoubtedly involved in ATL leukemogenesis, most leukemic cells do not express the viral protein Tax. Instead, cellular gene expression changes dominate homeostasis disorders of infected cells and characteristics of ATL. In this review, we summarize the state of the art of ATL molecular pathology, which supports the biological properties of leukemic cells. In addition, we discuss the recent discovery of two molecular hallmarks of potential generality; an abnormal microRNA pattern and epigenetic reprogramming, which strongly involve the imbalance of the molecular network of lymphocytes. Global analyses of ATL have revealed the functional impact of crosstalk between multifunctional pathways. Clinical and biological studies on signaling inhibitory agents have also revealed novel oncogenic drivers that can be targeted in future. ATL cells, by deregulation of such pathways and their interconnections, may become masters of their own destinies. Recognizing and understanding of the widespread molecular applicability of these concepts will increasingly affect the development of novel strategies for treating ATL.

## Introduction: Current Status of Adult T Cell Leukemia Research

**Figure 1:**
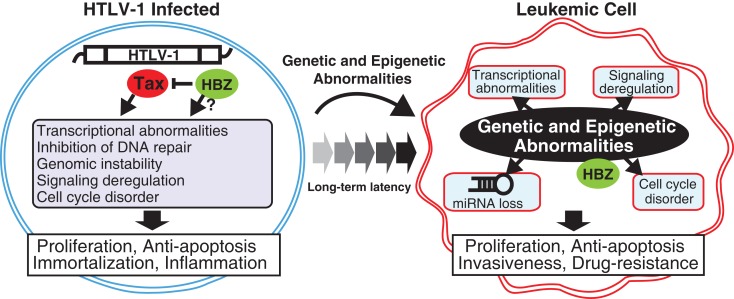
**Transition from HTLV-1-infected cell to transformed leukemic cell**. In early and latent clinical phases, the HTLV-1 Tax and HBZ mainly act as driving forces for the molecular hallmarks of infected cells. After a long-term latency, leukemic cells have acquired genetic and epigenetic abnormalities, which lead to deregulations of gene expression pattern, cell cycling, signaling activation, and miRNA expression. These molecular changes consequently induce cellular hallmark capabilities of ATL such as chronic proliferation, apoptotic resistance, multiple organ invasion, and drug resistance.

Adult T cell leukemia (ATL), which is derived from human T cell leukemia virus type 1 (HTLV-1)-infected CD4+ T cells, is an aggressive T cell leukemia/lymphoma with the worst poor prognosis (Yamaguchi and Watanabe, 2002). Endemic expansion of HTLV-1 infection and related ATL onset have been observed in Japan (Iwanaga et al., 2010). The diverse clinical features of ATL have led to its sub-classification into acute, lymphoma, chronic, and smoldering subtypes. ATL has a poor prognosis with a mean survival time of 13 months and is refractory to currently available combination chemotherapy (Yamada et al., 2001). It is therefore essential to develop a novel treatment strategy, in particular, a molecular targeting therapy. ATL is incurable because we do not have complete understanding of its molecular basis, leading to the lack of molecular targeting. Although HTLV-1 is an apparent causative agent of ATL, several studies have demonstrated that viral gene expression is rare, except for the expression of the HTLV-1 antisense gene product HBZ (Gaudray et al., 2002). Mounting evidence has shown that ATL does not contain the somatic mutant genes that can explain its aggressiveness. However, it is evident that ATL cells possess multiple deregulations of genome and gene regulation, namely the molecular hallmarks of ATL, which should be targeted (Figure 1). We believe that normal cells acquire a succession of molecular hallmarks as they progressively evolve into a neoplastic state and that the multistep process of human pathogenesis can be rationalized by the need of incipient cancer cells for acquiring traits that enable them to become leukemogenic and ultimately malignant. In this review, we first summarize the essence of each molecular hallmarks of ATL cells. Basic molecular analyses and next-generation global analyses of ATL samples have revealed the molecular traits of ATL. We address new developments that broaden the scope of the conceptualization and describe recent advances of science to acquisition of the molecular mechanistic underpinnings. Finally, we discuss the possibility and future direction of treating ATL.

## Molecular Hallmarks Controlled by Tax and HBZ

Since 1985, numerous excellent studies have indentified signaling abnormalities in HTLV-1-associated cells, mainly induced by HTLV-1 Tax. In general, T cell disorders include several deregulations of cellular processes that regulates the cell cycle, cell proliferation, and cell survival. Tax has been shown to disrupt all these cellular processes. The classical oncogenic function of Tax was first demonstrated in a study of cell cycle regulation. Tax was found to inhibit the cyclin-dependent kinase (CDK) inhibitor (CKI) CDKN2A (p16^INK4A^) by physical interaction (Suzuki et al., 1996). Several subsequent studies also revealed the mitogenic activity of Tax exerted through the stimulation of G1 to S phase transition (Akagi et al., 1996; Neuveut et al., 1998; Schmitt et al., 1998; Suzuki et al., 1999; Iwanaga et al., 2001; Haller et al., 2002; Liang et al., 2002). Tax also affects a cohort of cell cycle-related proteins, including CDKs, CDKN1A, CDKN1B, and CDKN2A, by regulating their expression or by physical interaction. Tax also participates in genetic damage (Jeang et al., 1990; Ressler et al., 1997; Kao and Marriott, 1999). Because of particular functions of Tax that can interact with many host factors (Interactome; Boxus et al., 2008; Simonis et al., 2012), Tax can activate several signaling pathways and lead to abnormal gene expression (a Tax-dependent molecular hallmark) and overproduction of several cytokines. Especially, IL-2 and its receptor are important for T cell activation. IL-2 signals through its receptor are primarily delivered by two molecular families, the Janus tyrosine kinases (JAKs) and signal transducers and activators of transcription (STATs), whose activation leads to lymphocyte proliferation. Tax can also activate the NF-κB and NFAT pathways responsible for the predominant expression of IL-2 and the IL-2 receptor (Ballard et al., 1988; Ruben et al., 1988; Hoyos et al., 1989; McGuire et al., 1993; Good et al., 1996). These findings implicate the IL-2–IL-2 receptor autocrine loop in ATL; however, several studies have shown that alterations of the loop alone are not sufficient to ensure the maintenance and proliferation of ATL cells because most Tax- or HTLV-1-immortalized T cells still require exogenous IL-2 and do not express detectable amounts of either IL-2 mRNA or protein (Akagi and Shimotohno, 1993; Chung et al., 2003; Chen et al., 2010). Similarly, IL-15 and its receptor and IL-13 and its receptor have been associated with Tax-expressing cells because similar to IL-2, they activate the STAT pathways (Azimi et al., 1999; Mariner et al., 2001; Chung et al., 2003; Wäldele et al., 2004). IL-6 is also transduced by Tax through the NF-κB pathway (Villiger et al., 1991; Muraoka et al., 1993). Because IL-6 mainly participates in inflammatory signaling, HTLV-1 infection can induce cytokine-dependent inflammation, which is frequently observed in ATL as well as HTLV-1-associated myelopathy/Tropical spastic paraparesis (HAM/TSP; Oh et al., 2011). OX40, a member of the TNF-receptor superfamily, is specifically expressed in HTLV-1-infected cells, whose expression is induced by the Tax-NF-κB pathway. Tax may play a role in leukemic cell infiltration in addition to cell adhesion *in vivo* (Imura et al., 1997). Tax affects not only the abovementioned signaling pathways but also the TGF-β pathway (Kim et al., 1990; Höllsberg et al., 1994; Arnulf et al., 2002; Lee et al., 2002). It has been recently shown that TGF-β signaling is activated by HBZ by binding with Smad 2/3 (Zhao et al., 2011). TP53 is the master regulator of the cell cycle that guards against DNA damage by inducing the transcription of several genes. Tax can inhibit TP53 functioning in multiple ways (Grassmann et al., 2005).

Strong NF-κB activation is the outstanding hallmark provided by Tax. NF-κB represents a family of inducible transcription factors that regulate diverse biological processes, including the growth and survival of both T cells and non-lymphoid cells. Transcriptional activation of genes such as several cytokines and apoptosis-resistance factors plays an important role in immunity. Tax acts as an intracellular stimulator of IKK by physical interaction, leading to persistent activation of NF-κB-mediated transcription. The Tax/IKK complex formation relies on the physical interaction between Tax and the IKK regulatory subunit IKKγ. The Tax/IKKγ interaction is required for recruiting Tax to the IKK catalytic subunits and for Tax-mediated IKK activation (Sun and Yamaoka, 2005). Recent studies have identified cellular proteins that are important for Tax-mediated NF-κB activation, such as NRP/Optineurin and TAX1BP1 (Journo et al., 2009; Shembade et al., 2011), and the ubiquitin-specific peptidase USP20 (Yasunaga et al., 2011). Subcellular localization of Tax also predominantly controls Tax-mediated NF-κB activation (Fryrear et al., 2009). Given that NF-κB governs the expression of a large array of cellular genes that control various cellular functions, the phenotypes of HTLV-1-infected cells are dominated by Tax-mediated abnormal activation.

Tax also activates several signaling pathways through key transcriptional factors such as CREB, SRF, and AP-1. It does not directly bind to promoter or enhancer DNA, however, disruption of these pathways causes serious gene expression disorders (Grassmann et al., 2005).

It should be also noted that HTLV-1 antisense product HBZ seems to be involved in leukemogenesis; its expression is sustained in leukemic cells. *In vitro* and *in vivo* studies have demonstrated that the growth-promoting activity of HBZ RNA may play an important role in oncogenesis by HTLV-I (Satou et al., 2006). Furthermore, transgenic expression of HBZ in CD4+ T cells induces T cell lymphomas and systemic inflammation in mice. HBZ directly induces *Foxp3* gene transcription, and the increased CD4+Foxp3+ T_reg_ cells in HBZ transgenic mice are functionally impaired, suggesting that the expression of HBZ in CD4+ T cells may be a key mechanism of HTLV-1-induced neoplastic and inflammatory diseases (Satou et al., 2011).

Taking together with these mounting evidences, Tax and HBZ undoubtedly contribute to leukemogenesis in HTLV-1-infected T cells. However, as a low rate of incidence, clinical observation implies that HTLV-1 itself does not have a strong capacity of leukemogenesis in contrast with other animal leukemia viruses.

## Chromosomal Changes and Gene Alterations in ATL

Tax is not expressed in most ATL cases because HTLV-1 provirus is substantially silenced by proviral defect and/or epigenetic mechanism (Tamiya et al., 1996; Koiwa et al., 2002; Taniguchi et al., 2005). However, leukemic cells possess very similar traits to Tax-expressing cells (Figure 1). The paradoxical truth, i.e., memory of Tax, still remains to be elucidated. Investigation of established ATL cell lines and primary ATL samples has led to the identification of the molecular hallmarks of leukemic cells, which may partially explain their malignant characteristics.

**Figure 2:**
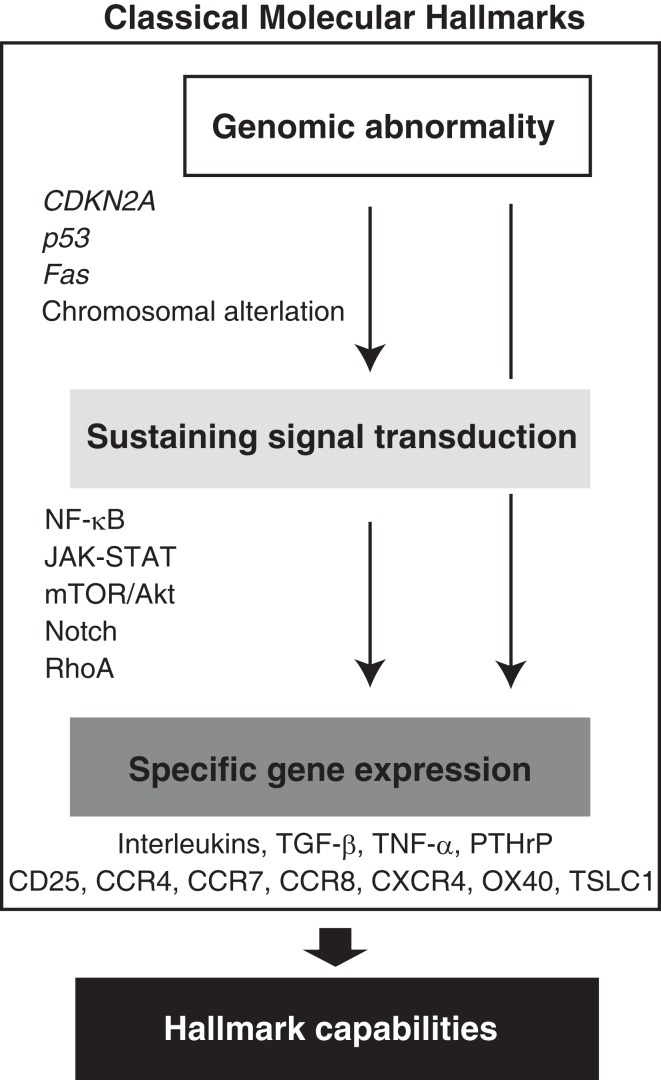
**Acquired molecular hallmarks of ATL**. From the beginning of ATL discovery, indicated molecular hallmarks have been suggested. Hierarchical regulation of the gene expression has been expected. Abnormal expressions of these cytokines and receptors as well as various proteins that act as anti-apoptotic factors or proliferation agents are responsible for the malignant phenotypes as hallmark capabilities.

From 1980s, chromosomal analyses of clinical cases were reported. Therefore, we know that ATL is characterized by various abnormal chromosomes. Kamada et al. (1992) reported that 96% of ATL cases had an abnormal chromosome pattern, suggesting that a genomic catastrophe underlies the clinical and molecular characteristics of ATL, which is consistent with all other cancers. In 2000s, global analysis has been available and whole genomic analysis could be challenged. Comparative genomic hybridization (CGH) revealed the genomic distinctions between the clinical subtypes; patients with aggressive ATL (acute and lymphoma) have a higher number of chromosomal imbalances, losses, and gains than those with indolent ATL (chronic and smoldering). Thus, genomic abnormalities are prognostic factors (Tsukasaki et al., 2001a). High-resolution analyses based on microarrays unveiled the genomic properties of ATL (Oshiro et al., 2006) and shed light on the genomic characteristics that affect the gene expression pattern (Figure 2).

In cancer cells, genetic mutations and deletions are generally observed in genes important for cell cycle regulation, cell survival, and cell proliferation. In ATL, the accumulation of genetic deletions in the gene encoding the cell cycle regulator CDKN2A have been reported (Hatta et al., 1995; Uchida et al., 1996). Southern blotting analyses of samples from large cohort studies (114 patients) also demonstrated that the *CDKN2* family (*p15* and *p16*) is lost in more frequently observed in the acute type (33.8%) than in the chronic type of ATL (5.4%; Yamada et al., 1997). Patients with these genetic deletions showed reduced survival compared with those without deletions. *CDKN2A* transcription is also regulated in an epigenetic manner. Abnormal DNA methylation has been during ATL progression (Nosaka et al., 2000).

Alterations of other cell cycle-regulating genes are unusual. Mutations of *CDKN1B* (*p27^Kip1^*) is rare in non-Hodgkin’s lymphoma and ATL (Morosetti et al., 1995). Mutations of *Rb* gene have also been observed in a few ATL patients (Hatta et al., 1997). Extensive analyses have revealed that 26–40% of ATL cases have a *p53* gene mutation, which is much less than that observed in other cancers (Nagai et al., 1991; Cesarman et al., 1992; Sakashita et al., 1992; Nishimura et al., 1995). Gene expression analysis of ATL samples showed that CDKN1A (p21^Cip1^) is commonly down regulated in ATL (Watanabe et al., 2010). In contrast, Tax activates p21^Cip1^ expression in HTLV-1-infected cells, which may be an important mechanism for stopping the host at the G1/S boundary and repairing damaged DNA before entering the S phase entry (Cereseto et al., 1996; de La Fuente et al., 2000). Although *p21^Cip1^* transcription may be activated by the Tax–CREB pathway in infected cells, it seems to be epigenetically silenced in several cancers, including ATL. Thus, these gene alterations and related expression patterns are important molecular hallmarks of ATL as well as other cancers. Of note, no ATL cell has been shown to have both mutations, suggesting that either *p16* or *p53* mutation may be sufficient to promote the more aggressive phenotype of leukemic cells (Tawara et al., 2006). In addition, as many ATL patients carry a wild-type *p53*, TP53 activation by antagonists such as Nutlin-3a may be a promising strategy for ATL therapy (Hasegawa et al., 2009). The ability to resist apoptotic cell death is conferred by *Fas* gene mutation (Tamiya et al., 1998; Maeda et al., 1999). Indeed, Fas-negative ATL cells are resistant to adriamycin-induced apoptosis *in vitro*, which is consistent with the finding that ATL in this case is resistant to chemotherapy.

Single nucleotide polymorphism (SNP) is another possible cause for developing ATL. TNF-α polymorphism may be associated with increased susceptibility to development of ATL in HTLV-1 carriers (Tsukasaki et al., 2001b). An immunological study also suggested that the HLA haplotype is related to ATL progression (Yashiki et al., 2001). Currently, whole SNP can be detected by next-generation sequencing and other array-based techniques. Global understanding of SNPs in ATL patients may provide us with the genetic basis of the familial clustering of ATL.

By utilizing global survey techniques for analyzing the gene copy number, several research groups have detected specific gene copy number patterns in ATL. CGH methods first demonstrated gains at 14q32 and 2p16-22 in ATL cell lines (Ariyama et al., 1999). Fluorescence *in situ* hybridization (FISH) analysis with several yeast artificial chromosome (YAC), BAC, and PAC clones also mapped the breakpoints in ATL cell lines with 6q aberrations (Tagawa et al., 2002). Clinical subtype-specific genomic alterations in aggressive ATL were also identified by array-based CGH (Oshiro et al., 2006). The lymphoma type of ATL had significantly more frequent gains at 1q, 2p, 4q, 7p, and 7q and losses at 10p, 13q, 16q, and 18p, whereas the acute type showed a gain of 3/3p. *CARMA1* is also a possible target gene for 7p22 amplification in the lymphoma type but not in the acute type of ATL. In contrast, BCL11B is expressed in the acute type of ATL, regardless of 14q32 gain/amplification; however, there is no or low expression of the gene in the lymphoma type of ATL. Taken together, these findings suggest that the acute and lymphoma types of ATL are genomically distinct. The physiological molecular hallmarks of ATL have been identified by integrating the gene copy number and gene expression analyses findings, and these are described below.

## Global Gene Expression Analyses of ATL

Genetic alterations and other modulations enhance specific gene expression signatures. Classically, based on a comparison between normal T cells and ATL cell lines as well as primary leukemic cells, ATL has been shown to have a distinct gene expression pattern that may reflect its clinical pathogenesis. In addition, global expression analyses, mainly based on microarray, have identified emerging molecular hallmarks that can possibly be targeted in ATL therapies.

First, Harhaj et al. (1999) performed gene array analysis of HTLV-1-immortalized cells and reported up- and downregulated genes. Ruckes et al. (2001) performed suppressive subtractive hybridization that enabled the isolation of novel sequences derived from unknown genes. They identified a number of genes linked to Tax transformation and ATL leukemogenesis. Systematic comparison of gene expression between cultured cells from patients with acute ATL and that of stimulated peripheral T lymphocytes revealed 346 cDNA clones, which included the genes encoding IL-2 receptor α chain and p21^Cip1^. They also included a dual-specific protein phosphatase (PAC1), an interferon-inducible factor (ISG15), a basic helix–loop–helix transcription factor (DEC-1), and the secreted anti-apoptotic chemokine I-309. Pise-Masison et al. (2002) also reported the results of analyses performed using an Affymetrix Hu6800 GeneChip. They identified 763 genes with differentially regulated expression in HTLV-1 cell lines.

Tsukasaki et al. (2004) directly analyzed the gene expression patterns of fresh ATL samples (three acute types and one chronic type) using high-density oligonucleotide DNA arrays. A total of 203 genes that included ribosomal proteins, proteasome subunits, translation factors, immunophilins, heat shock proteins, and genes important for DNA replication were found to be upregulated in ATL cases, and 91 genes were downregulated. Sasaki et al. (2005) also determined the expression profiles of more than 12,000 genes in eight cases of the acute type of ATL using microarray analysis, and they found that 192 genes were upregulated more than twofold compared with healthy CD4+ and CD4+/CD45RO+ T cells. In particular, the expression of tumor suppressor in lung cancer 1 (TSLC1), caveolin 1, and prostaglandin D2 synthase was increased by more than 30-fold. Interestingly, comparison of gene expression between established cell lines with or without HTLV-1 infection led to the identification of the specific HTLV-1-related cell surface marker CD70, which is also expressed in freshly isolated leukemic cells (Baba et al., 2008).

Integrated analyses of the genome and its expression revealed more detailed data for the acquisition of molecular and physiological information. Choi et al. (2007) isolated CD4+ T cells from ATL patients and profiled their gene expression using DNA microarrays containing >44,000 probe sets. Changes in the chromosomal copy number were examined for 24 cell specimens using microarrays harboring approximately 50,000 probe sets. Coordinated analysis revealed a novel molecular target, the HGF–MET pathway. Ligation of MET by increased plasma HGF levels may confer a growth advantage on ATL cells through the phosphatidylinositol 3-kinase (PI3K) and Ras pathways.

We performed further global integrated analyses of samples from ATL patients using mRNA expression array (*n* = 52), microRNA (miRNA) microarray (*n* = 40), and DNA copy number analysis using SNP-based array (*n* = 168; Yamagishi et al., 2012). Each analysis revealed many novel molecular characteristics of ATL (data available in the Gene Expression Omnibus database). Analysis of this massive dataset derived from primary ATL samples led to the conclusion that genetic and epigenetic imbalances are involved in ATL leukemogenesis through miRNA and the signaling pathways responsible for leukemic cell survival, proliferation, and invasiveness. Taken together, these findings clearly indicate that disrupted gene expression is a molecular hallmark of ATL. Diverse abnormalities have been found in each of these comprehensive studies; however, several gene alterations and other critical events have been commonly implicated as determinants of the gene expression pattern. Abnormal expression of different cytokines, their receptors, and various proteins that act as anti-apoptotic factors or proliferation agents is responsible for malignant phenotypes as hallmark capabilities (Figure 2). From the spreading intelligence, we have to acquire the genuine molecular targets harboring in the bare bones of ATL.

## Signaling Networks in ATL: Main Driving Forces

The cellular complexity of signaling networks confers robustness to specific gene expression and biological functions. This is supported by the fact that the inhibition of a signaling pathway can strictly suppress abnormal gene expression. The functional characteristics of ATL cells, such as chronic proliferation, abnormal survival, and penetrating multiple organs, is supported by aberrant gene expression, as described above.

**Figure 3:**
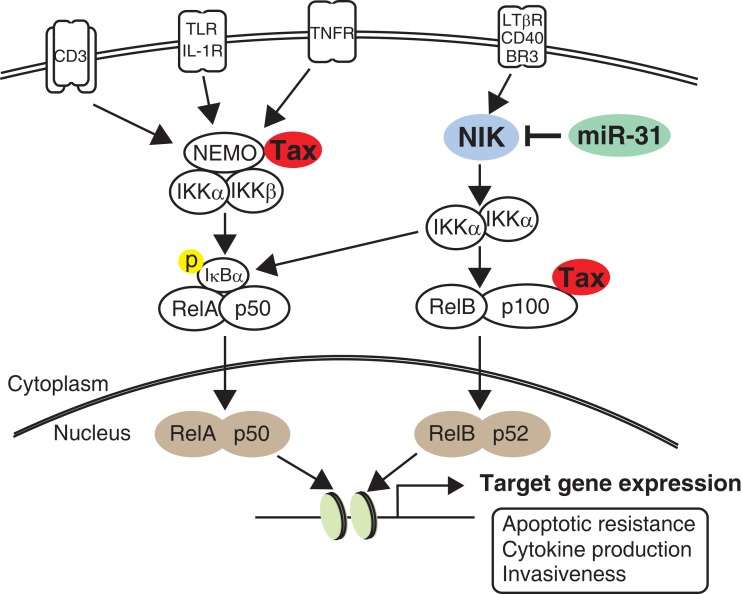
**NF-κB signaling pathway in ATL**. Compared to normal T cell, HTLV-1-infected cell shows intense activity of NF-κB pathway through the Tax interactome. In contrast, leukemic cell sustains strong NF-κB activity without Tax by acquisition of miR-31-dependent NIK expression. Activation of the non-canonical pathway ultimately joins canonical pathway, resulting in transactivation of numerous target genes important for cell survival, cytokine production, and invasiveness.

Activation of NF-κB signaling is the most outstanding hallmark of both HTLV-1-infected cells and leukemic cells. Abnormal activation and deficient negative control of the NF-κB pathway strongly contributes to prolonged survival, proliferation, and invasiveness, all of which are observed in ATL cells. Ruben et al. (1988) found that Tax can activate IL-2 receptor gene expression in a NF-κB-dependent manner. Following this, several studies clearly demonstrated that Tax can activate NF-κB activity through a multistep (Sun and Yamaoka, 2005). Strong and persistent activation of NF-κB signaling was then demonstrated in leukemic cell lines that did not express Tax (Mori et al., 1999). In transformed ATL cells, both the canonical and non-canonical NF-κB pathways are persistently activated because NF-κB inducing kinase (NIK) is aberrantly expressed in ATL (Saitoh et al., 2008). NIK plays a central role in non-canonical NF-κB signaling by IKKα phosphorylation (Thu and Richmond, 2010). Very recently, we found a novel linkage between NF-κB activation and miRNA deregulation. Comprehensive gene expression analysis and *in vitro* experiments revealed that miRNA-31 (miR-31) can regulate NIK expression through the 3′ untranslated region (UTR). In ATL cells, miR-31 expression is genetically and epigenetically silenced, which in turn induces constitutive NF-κB activation through NIK expression (Figure 3; Yamagishi et al., 2012). Because current evidence clearly indicates that miR-31 dominates NF-κB activity in T cells, manipulating cellular miR-31 levels may be a novel molecular approach to reduce NF-κB activity and induce apoptosis.

Numerous efforts have demonstrated that NF-κB inhibitory agents may be suitable for treating ATL. Inhibitors such as bortezomib (Satou et al., 2004), Bay 11-7082 (Mori et al., 2002), DHMEQ (Watanabe et al., 2005), ACHP (Sanda et al., 2006), and IMD0354 (Uota et al., 2012) have the ability to kill leukemic cells. Fludarabine, a purine analog that has significant activity in B cell malignancies, also inhibits the NF-κB activity in ATL (Nishioka et al., 2007). Tax degradation by arsenic trioxide and IFN-α treatments is also useful for inhibiting NF-κB in HTLV-1-infected cells (El-Sabban et al., 2000). In the near future, gene manipulation for example by siRNA targeting NIK may be a promising strategy for inhibiting NF-κB activity with strict specificity (Yamagishi et al., 2012). Because NF-κB prevention showed good results in a xenograft model of cell lines with or without Tax (Dewan et al., 2003; Watanabe et al., 2005; Ohsugi et al., 2007a), molecular targeting therapy based on the NF-κB pathway is a promising new ATL treatment. Of note, NF-κB inhibition by DHMEQ can also remove virus-carrying cells from carrier peripheral blood mononuclear cell (PBMC) samples (Watanabe et al., 2005).

Why is NF-κB important for ATL cell survival? One of the target genes is BCL-XL, which is expressed when NF-κB is stimulated. In HTLV-1-infected cell lines, BCL-XL is expressed through the Tax-NF-κB pathway. Interestingly, fresh ATL samples show BCL-XL overexpression (Nicot et al., 2000). Given that BCL-XL is a principal anti-apoptotic protein as a BCL-2 family member, persistent BCL-XL expression is one of the molecular capability of apoptotic resistance, which may contribute to clinical chemoresistance. Indeed, inhibition of the NF-κB pathway by NIK depletion leads to impaired BCL-XL levels and apoptotic cell death in ATL (Yamagishi et al., 2012). In addition, a recent study has implicated that both BFL1 (BCL2A1) and BCL-XL are responsible for ATL cell survival. BFL1 expression is regulated by Tax and HBZ through the NF-κB and AP-1 pathways, respectively (Macaire et al., 2012).

The JAK–STAT pathway, which is universal and essential to cytokine receptor signaling, is also one of the best understood signal transduction cascades in several cell types, including ATL cells. The JAK–STAT pathway mediates signaling by cytokines, which control cell survival, proliferation, and differentiation. Constitutive JAK activation leads to persistent activation of STAT transcription factors, and several cancers exhibit constitutive STAT activation in the absence of JAK- or STAT-activating mutations. JAK–STAT activation in HTLV-1-infected cells was first reported in 1995 (Migone et al., 1995). Takemoto et al. (1997) demonstrated that STAT-3 and STAT5 are activated in freshly isolated ATL cells. However, functional redundancy was also reported. Blockade of the JAK3–STAT5 pathway by AG490 failed to inhibit the proliferation of HTLV-1-transformed T cell lines, despite activation of the pathway (Kirken et al., 2000). Recently, a JAK2–STAT5 inhibitor, AZ960, was shown to have an anti-proliferative effect on HTLV-1-infected and ATL cell lines (Yang et al., 2010). However, which gene is transactivated by STAT remains unknown. The biological relevance of the JAK–STAT pathway in ATL remains to be elucidated.

## Emerging Signaling Pathways and Inhibitory Agents

In addition to the abovementioned pathways, several signaling pathways, including the AP-1, NFAT, and CREB pathways, may be involved in the functional characteristics of ATL (Hall and Fujii, 2005). These are activated by Tax. Leukemic cells may have certain activities in each of these pathways.

The RhoA family also participates in Tax-mediated immortalization. A recent study employing a proteomic approach to identify Tax-binding proteins in a HTLV-1-infected T cell line identified direct interactions between Tax and several small GTPases, including RhoA, Rac1, and Cdc42, all of which are involved in a wide variety of cellular processes, including cytoskeleton organization and transcriptional activation (Wu et al., 2004). Recently, our global gene expression analysis as well as miRNA study strongly suggested that RhoA is overexpressed in ATL cells. A decreased miR-31 level may contribute to RhoA expression and migration activity (Yamagishi et al., 2012). The gene expression signature determined from patient samples could identify the additional signaling pathways involved in leukemogenesis (authors’ unpublished data).

Recently, several studies have demonstrated that ATL shows emerging signaling activation, which is well established as oncogenic signaling in other types of cancer. Typically, the Notch pathway, which is the signaling pathway implicated in T cell acute lymphoblastic leukemia (T-ALL), is also activated in ATL (Pancewicz et al., 2010). More than 30% of ATL cases show activating mutations in *NOTCH1*, leading to reduced CDC4/Fbw7-mediated degradation and stabilization of the intracellular cleaved form of Notch1 (ICN1). Inhibition of Notch signaling by a γ-secretase inhibitor reduced tumor cell proliferation and tumor formation in ATL-engrafted mice. It is therefore suggested that activated Notch may be important for ATL pathogenesis.

TGF-β signaling is also reportedly important. Increased TGF-β production was observed in freshly isolated ATL cells, and this was at least partially mediated by AP-1 (Niitsu et al., 1988; Kim et al., 1990). Nevertheless TGF-β could inhibit the growth of normal T lymphocytes, HTLV-1-infected cells were resistant to this inhibition (Höllsberg et al., 1994). In ATL cells, transcription factor 8 (TCF8) is frequently disrupted by several mechanisms, primarily by epigenetic dysregulation. TCF8 mutant mice frequently develop invasive CD4+ T cell lymphomas *in vivo*. Downregulation of TCF8 expression in ATL cells *in vitro* is associated with resistance to TGF-β1, suggesting that escape from TGF-β1-mediated growth inhibition is important in the pathogenesis of ATL (Hidaka et al., 2008). The same research group also reported that ZEB1 downregulation and Smad7 overexpression contribute to resistance to TGF-β1-mediated growth suppression in ATL (Nakahata et al., 2010). ZEB1 plays a critical role in regulating the epithelial–mesenchymal transition (EMT) in several solid cancers. The general function of ZEB1 in lymphocytes, particularly in association with leukemogenesis and TGF-β signaling, is a very intriguing issue.

If a chemical drug that can inhibit cellular signaling can induce apoptosis specifically, it is probable that leukemic cell survival is supported by the targeted signaling. Recent studies of inhibitors have indicated that mTOR signaling may be involved in ATL. mTOR is a serine/threonine-specific protein kinase that is located downstream of the PI3K/Akt pathway. Deregulation of mTOR signaling is implicated in a range of cancers due to its roles in cell survival and proliferation, protein synthesis and breakdown, membrane trafficking, and protein kinase C signaling (Zoncu et al., 2011). Ikezoe et al. (2007) used several inhibitors to show that the PI3K/Akt/mTOR pathway is activated in ATL cell lines as well as fresh ATL cells. In addition, a resent study reported that the mTOR complex 1 (mTORC1) inhibitor everolimus has a dramatic inhibitory effect on the growth of HTLV-1-positive and HTLV-1-negative malignant T cells, whereas normal resting or activated T cells are resistant to it (Darwiche et al., 2011). Furthermore, in addition to HTLV-1-infected cells in which Tax activates the Akt pathway (Liu et al., 2001), transformed leukemic cells in which Tax is not expressed may have some Akt activity. The most important point is how these pathways are constitutively activated in ATL. The functional importance of this pathway and its therapeutic potential will be addressed in the near future.

In the context of inhibitory agents, a higher response rate following azidothymidine/interferon α (AZT/IFNα) treatment of ATL patients has been reported in several human trials (Gill et al., 1995; Bazarbachi et al., 2010). AZT treatment of ATL patients inhibits telomerase activity and results in progressive telomere shortening and increased p14^ARF^ expression. A functional relationship between *p53* and sensitivity to AZT has also been suggested (Datta et al., 2006). Ritonavir, developed as a protease against HIV-1, also has an anti-ATL effect. Ritonavir decreases NF-κB activity linked to the inhibition of IκBα phosphorylation and induces the apoptosis of ATL cells. In addition, it very efficiently prevents tumor growth and leukemic infiltration in various organs of NOG mice when administered at the same dose as that used in the treatment of patients with AIDS (Dewan et al., 2006). Together, several signaling networks are deregulated in leukemic cells, and these are a specific molecular feature of ATL (Figure 2). At present, signal interception is the most effective strategy to treat ATL. At the same time, the occurrence of incidental side effects should be carefully considered because these signaling pathways are essential for normal cell function and survival.

## Cytokines Production and ATL

CD4+ T cells play a central role in the immune response by controlling cells such as B cells, dendritic cells, and cytotoxic cells and their responses through various cytokines. Deregulation of the associated signaling pathways leads to abnormal gene expressions, including that of several cytokines. ATL has been implicated in the production of various cytokines, including IL-1 (Wano et al., 1987), TGF-β (Niitsu et al., 1988), TNF-α, IFN-γ, GM-CSF (Yamada et al., 1996), and PTHrP (Watanabe et al., 1990). Especially, an elevated serum C-terminal PTHrP level is a characteristic marker of the HTLV-1 carrier status, and the determination of this level in ATL patients could be useful for assessing the prognosis (Yamaguchi et al., 1994). SCID mice model of ATL also showed clearly elevated serum levels of calcium and C-terminal PTHrP, resulting in the development of hypercalcemia (Takaori-Kondo et al., 1998). The high frequency of hypercalcemia is one of the notable clinical characteristics of ATL, in particular, the aggressive types of ATL (Kiyokawa et al., 1987). Besides PTHrP, which plays an important role in bone resorption by stimulating osteoclasts, abnormal expression of the RANK ligand (RANKL) has also been demonstrated in ATL with hypercalcemia (Nosaka et al., 2002). Recent studies have revealed that a central region of HTLV-1 gp46 acts as an antagonist for osteoprotegerin and leads to hypercalcemia (Sagara et al., 2007, 2009).

SCID mice engrafted with cells from Tax-transgenic mice that develop lymphoma produced TNF-α, PDGF-BB, sICAM-1, and sVCAM-1 as factors that may contribute to high levels of organ infiltration (Watters et al., 2010). PDGF, in particular, the BB isoform, is a well-known potent osteotropic factor that stimulates the osteoclasts and osteoblasts functions (Yi et al., 2002). High IL-2 production was not observed in previous ATL studies or in our microarray data (Yamagishi et al., 2012). Nevertheless, IL-2 is an HTLV-1-induced cytokine associated with the NF-κB pathway (Hoyos et al., 1989). In contrast, receptor subsets for IL-2 (IL2Rs) are generally overexpressed in ATL cells.

Interestingly, smoldering/chronic ATL PBMCs spontaneously proliferate *ex vivo* in an IL-12-, IL-9-, and IL-15-dependent manner, whereas acute type ATL PBMCs do not proliferate or proliferate independent of cytokines. Furthermore, purified leukemic cells from indolent ATL cases produce IL-2/IL-9 and the downstream JAK–STAT pathway is activated. Thus, autocrine/paracrine cytokine stimulation of leukemic cell proliferation may occur in patients with smoldering/chronic ATL (Chen et al., 2010).

## Cell Surface Markers and Their Functions

IL-2 receptor α (CD25) was the first marker of ATL and HTLV-1-infected cells. CD25 expression is dependent on NF-κB activity (Ruben et al., 1988). Global gene expression analysis has also validated the high CD25 mRNA level in ATL patient samples (Yamagishi et al., 2012).

Chemokines and their receptors mainly function in the migration and tissue localization of lymphocytes. The expression of the following ATL-specific chemokine receptors has been identified: CCR4 (Yoshie et al., 2002), CCR7 (Hasegawa et al., 2000), CCR8 (Ruckes et al., 2001), and CXCR4 (Twizere et al., 2007). In addition, other cell surface proteins such as OX40 (Imura et al., 1997) and TSLC1 (Sasaki et al., 2005) are highly expressed in ATL cells and are thus the molecular hallmarks of ATL; they may also participate in leukemogenesis. For example, TSLC1, a well-known tumor suppressor in various carcinomas, is overexpressed in ATL. The cytoplasmic domain of TSLC1 directly interacts with the PDZ domain of TIAM1 and induces the formation of lamellipodia through Rac activation in HTLV-1-transformed and ATL cell lines. TIAM1 may integrate signals from TSLC1 to regulate the actin cytoskeleton through Rac activation (Masuda et al., 2010).

Some ligands are also expressed by ATL cells; therefore, autocrine/paracrine stimulation is implicated. Tax develops a strategy based on the activation of the SDF-1α/CXCR4 axis in infected cells (Twizere et al., 2007). In Tax-transgenic mice and their transplantation model, AMD3100, a CXCR4 antagonist, inhibits the infiltration of lymphomatous cells into tissues *in vivo*, indicating the involvement of the SDF-1α/CXCR4 interaction in leukemic cell migration (Kawaguchi et al., 2009). CCR4 expression is clinically considerable. The defucosylated anti-CCR4 monoclonal antibody KW-0761 induces CCR4-specific antibody-dependent cellular cytotoxicity (ADCC) against CCR4-positive ATL cells. In view of its molecular functions, CCR4 expression may also account for the frequent infiltration of ATL cells into the skin and lymph nodes (Yoshie et al., 2002). Specific surface markers are therefore worthy of attention to identify concentrations of leukemic cells as well as minor infected cells in asymptomatic carriers. A recent study reported the development of a new method for concentrating leukemic cells by multi-color flow cytometry. The majority of leukemic cells are included in the CD4+, CD3-dim, and CD7-low subpopulations (Tian et al., 2011). Consequently, characteristic expression of cytokines and their receptors is clearly required for leukemic cell behavior, which in turn may be used as landmarks and/or therapeutic motifs.

## New Paradigm from miRNA

According to the summary of previous ATL studies described above, we can fight ATL to a certain extent. However, we cannot cure ATL because of relapse with multidrug resistance, immunodeficiency, and strong invasiveness. In addition to the previously proposed molecular hallmarks (Figure 2), we urgently need a conceptual advance that can promote understanding of the source of disrupted gene expression. Indeed, in the course of our remarkable progress in researching ATL and other malignancies, new observations have helped in clarifying and modifying the original formulations of the hallmark capabilities.

One of the most significant recent advances in biomedical research has been the discovery of the 22-nt-long class of non-coding RNA designated miRNA that posttranscriptionally regulates gene expression by binding to the target mRNAs. miRNA is expressed by all metazoans and plants, as well as by several DNA viruses; it regulates cellular processes such as development, differentiation, growth, homeostasis, stress responses, apoptosis, and immune activation (Esquela-Kerscher and Slack, 2006). In ATL filed, some studies have been reported, and several miRNA aberrations have been identified in HTLV-1-infected cells and ATL samples.

Pichler et al. (2008) first identified abnormal miRNA expression in HTLV-1-infected cells. They explored the interconnections between HTLV-1 and cellular miRNAs by using several HTLV-1-transformed cell lines. miR-21, miR-24, miR-146a, and miR-155 were found to be upregulated and miR-223 was found to be deregulated in HTLV-1-infected cells. In particular, miR-146a expression was directly stimulated by Tax through the NF-κB pathway. *In silico* analysis predicts that many candidate genes may be deregulated by miRNA changes (Pichler et al., 2008).

Yeung et al. (2008) performed miRNA microarray analysis of 327 well-characterized human miRNAs in 7 HTLV-1-related cell lines and four acute ATL patient samples. They found that miR-18a, miR-93, and miR-130b were overexpressed in ATL samples. Of note, these miRNAs were also upregulated by PHA-mediated T cell activation. Tumor protein p53 inducible nuclear protein 1 (TP53INP1) is a gene targeted by one of miR-93 and miR-130b, and reduced TP53INP1 expression mediated by miRNA upregulation contributes to cell proliferation and survival (Yeung et al., 2008).

Bellon et al. (2009) also reported the result of miRNA array analysis of 7 ATL samples and normal PBMC and CD4+ T cells and revealed that miR-150, miR-155, miR-223, miR-142-3p, and miR-142-5p are upregulated, whereas miR-181a, miR-132, miR-125a, and miR-146b are downregulated in ATL. They discussed that miRNAs involved in normal hematopoiesis and immune responses are profoundly deregulated in ATL tumor cells *ex vivo* (Bellon et al., 2009).

Each of these studies has identified interesting miRNAs that are deregulated in ATL-related cells; however, no identical miRNA patterns have been observed. The amount of cellular miRNAs may be susceptible to various environmental conditions such as transcriptional activity, maturation processing, and epigenetic regulation. The end results appear to be affected by the methodology employed and the conditions and types of samples used. Very recently, we established global gene expression analyses of a large cohort ATL study that included analyses of mRNA expressions, miRNA levels, and genomic copy number (Yamagishi et al., 2012). A strict threshold (*p* < 1 × 10^−5^) and two-dimensional hierarchical clustering analysis revealed 61 miRNAs with significantly altered expression levels in ATL cells (*n* = 40) compared with control CD4+ T cells (*n* = 22). It is most important that primary ATL samples show global miRNA downregulation, similar to observations in other cancer researches (Lu et al., 2005; Gaur et al., 2007). Fifty-nine of the 61 miRNA (96.7%) showed decreased expression in ATL. The amount of cellular miRNA may be susceptible to various environments such as transcriptional activity, maturation processing, and also epigenetic regulation. Among them, miR-31 is the most profoundly repressed miRNA in all ATL individuals (fold change, 0.00403). It is a known tumor suppressor that may also be associated with metastatic breast cancer (Valastyan et al., 2009). Other downregulated miRNAs found in ATL patients may also be involved in the hallmark capabilities of ATL, since they are uniformly decreased in tested ATL samples and each miRNA may regulate a large number of genes.

Several predictions and experimental approaches have defined a novel miR-31 target gene, MAP3K14 (also called NIK), which is a persistent NF-κB activator in various malignancies, including B cell lymphoma (Pham et al., 2011), multiple myeloma (Annunziata et al., 2007), breast cancer (Yamamoto et al., 2010), pancreatic cancer (Nishina et al., 2009), and ATL (Saitoh et al., 2008). Interestingly, all these malignancies have low miR-31 levels. Manipulation of the miR-31 level clearly indicated that the miR-31 level was negatively correlated with cellular NF-κB activity. Importantly, enforced miR-31 expression in B cells attenuated both BAFF and CD40L-mediated NIK accumulation and subsequent canonical and non-canonical NF-κB signaling. As discussed above, NF-κB activity dominates the regulation of apoptosis and subsequent cell survival. Induced miR-31 expression or NIK knockdown reduces apoptotic resistant proteins such as BCL-XL and XIAP, resulting in strong apoptosis in ATL cell lines as well as in primary leukemic cells from ATL patients (Yamagishi et al., 2012). Several lines of evidence definitively support two notions: (1) miR-31 acts as a tumor suppressor in T cells and (2) NIK-regulated NF-κB is of pivotal importance to cancer cell survival (Uribesalgo et al., 2012).

**Figure 4:**
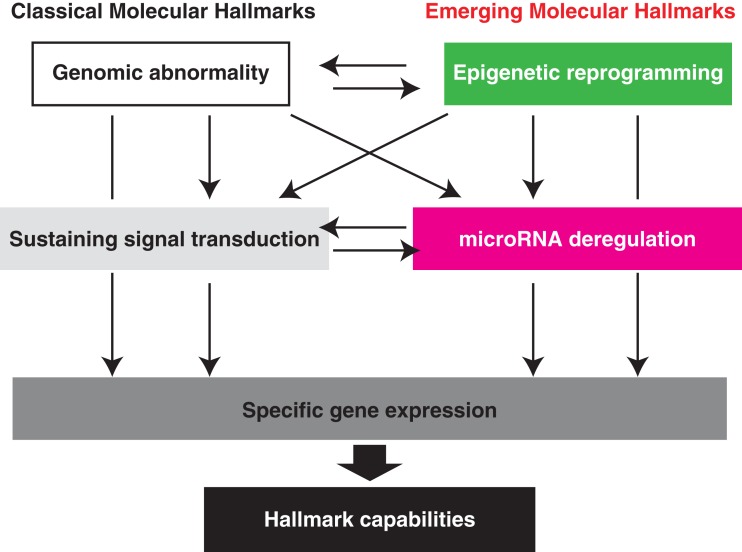
**Emerging molecular hallmarks, microRNA deregulation and epigenetic reprogramming**. An increasing body of research suggests that two additional molecular hallmarks are involved in the pathogenesis of ATL. In addition to the genomic abnormality, epigenetic imbalance widely governs the downstream molecular capabilities. Deregulation of the cellular miRNA levels directly influence hundreds of genes expression. Importantly, cross talking among each category can attain more complex gene regulatory network that is indispensable for exercise of various functions at appropriate timing.

The fact that deregulated miRNA expression predominates NF-κB activity is a conceptual advance. Regulation of global miRNA downregulation and each regulatory network may shed light on our understanding of the next-generation molecular hallmarks of ATL and of molecules suitable for therapeutic targeting (Figure 4). Since a single miRNA can regulate the expression of multiple genes, pleiotropic miRNA may have potential as molecular therapy. Profound miR-31 loss is a characteristic of ATL; however, decreased miR-31 expression seems to be commonly observed in various malignancies. The regulatory mechanism of miR-31 had not been identified until our discovery. In general, down modulation of gene expression is coordinated by some contents of transcriptional factors and an epigenetic regulatory mechanism.

## Epigenetic Deregulation Observed in ATL

Technological advances in genomics and epigenomics have supplied new methods to distinguish one cell type from another. The epigenetic code consists of the combined on–off states of hundreds of genes, which coordinately dictate cellular identity and function. Increasing attention is being paid to global regulatory factors and molecular mechanisms by which control gene transcription. This genome programming operates fundamentally through DNA methylation, histone chemical modification, and protein complex binding in these environments.

Cancer-associated epigenetic reprogramming has been suggested because DNA methylation is a transcriptional regulator. In ATL, no attempt has been made to determine global epigenetic statements that can explain deregulated gene expression. Analysis of epigenetic factors such as DNA methylation and related gene silencing has been reported, particularly in some tumor suppressor genes such as the CDK inhibitor family.

The CpG island of *CDKN2A* gene is more frequently methylated in fresh tumor cells isolated from patients with acute ATL (47%) or lymphoma type ATL (73%) than in fresh tumor cells isolated from patients with chronic (17%) and smoldering (17%) ATL, which are relatively less malignant (Nosaka et al., 2000). No *CDKN2A* gene is methylated in asymptomatic carriers or uninfected individuals. A possible inverse correlation between *CDKN2A* mRNA expression and gene methylation status is suggested. Methylation-specific polymerase chain reaction (MSP) also suggested the presence of an additional DNA methylation in *CDKN2B* gene (20%; Hofmann et al., 2001). In addition to the cell cycle regulators, multifunctional factors involved in cell proliferation, differentiation, and apoptosis, e.g., bone morphogenetic protein (BMP) is deregulated by aberrant DNA methylation in malignant lymphomas (Daibata et al., 2007) and also ATL (Taniguchi et al., 2008). Above all, the BMP6 promoter is hypermethylated in ATL: acute (96%), lymphoma (94%), chronic (44%), and smoldering (20%). BMP6 promoter methylation seems to be a common epigenetic event at later stages of ATL. The adenomatous polyposis coli (APC) gene is also a tumor suppressor, and its mRNA level is at least partially regulated by DNA methylation (Tsuchiya et al., 2000). In ATL, 48% of primary samples have methylated promoter DNA in the APC region (Yang et al., 2005).

The methylated CpG island amplification/representational difference analysis method revealed 53 aberrantly hypermethylated DNA sequences in ATL (Yasunaga et al., 2004). Among them, kruppel-like factor 4 (KLF4) and early growth response 3 (EGR3) were found to be responsible for apoptotic resistance in ATL cell lines, implicating that DNA methylation is involved in leukemogenesis. Abnormal DNA demethylation may also be involved. MEL1S, an alternatively spliced form of MEL1, is frequently expressed in ATL cells because of DNA hypomethylation at an alternative transcriptional start site. Aberrant MEL1S expression is associated with dysregulation of TGF-β-mediated signaling (Yoshida et al., 2004). Thus, altered DNA methylation pattern, including DNA demethylation, is one of the molecular hallmarks of ATL linking leukemogenesis to gene transcription control.

Histone modifications such as histone acetylation and specific methylations confer dynamic exchanges of transcription. Although a global survey of histone modification in ATL (such as by ChIP-on-chip analysis) has not been reported, experimental evidence with epigenetic drugs strongly suggests that epigenetic reprogramming is the background of the molecular hallmarks of ATL. Histone deacetylase (HDAC) inhibitors effectively inhibit the proliferation of several cancers (Spiegel et al., 2012) as well as that of HTLV-1-infected cell lines and primary ATL samples (Nishioka et al., 2008). Analysis of signaling cascades suggested that HDAC inhibition can block nuclear translocation of NF-κB components. Paradoxically, another study implicated that the HDAC inhibitors can actively modulate the NF-κB pathway through RelA acetylation (Chiechio et al., 2009). Anyway, abnormal histone deacetylation may be involved in cell survival and cell cycle regulation in ATL cells.

Histone acetylation and DNA methylation actually cooperate in regulating a cohort of genes during multiple processes of leukemogenesis. For example, thioredoxin-binding protein-2 (TBP-2) expression is lost during the transformation step in HTLV-1-infected T cells (Nishinaka et al., 2004). TBP-2 seems to play a crucial role in the growth regulation of T cells. Sequential treatment with a DNA methylation inhibitor, 5-Aza-dC, and an HDAC inhibitor can restore the TBP-2 expression, suggesting that loss of TBP-2 expression is caused by both DNA methylation and histone deacetylation in transformed infected cell lines (Ahsan et al., 2006).

Besides acetylation, the N-terminus of histone proteins contains several residues that can be methylated. Integrated histone modification consequently decides the degrees of chromatin condensation and subsequent transcriptional sensitivity. Trimethylation of the histone H3 Lys27 (H3K27me3) mark plays a central role in the repression of transcription, mainly in the euchromatin region. The Polycomb family is a master regulator of the H3K27me3 level by inducing and maintaining the histone mark. Progress over the past decade has defined two main protein complexes: Polycomb repressive complex 1 (PRC1) and PRC2, with fundamental roles in Polycomb-mediated gene silencing (Schuettengruber et al., 2007). PRC2 methylates the histone histone 3 lysine 27 (H3K27). PRC1 is commonly viewed as an important, direct executor of silencing at target genes. Although H3K27 methylation is a key chromatin mark, there is ongoing debate about its molecular consequences. In the context of cancer research, deregulation by the Polycomb family confers a specific gene expression pattern responsible for chronic proliferation, survival, peculiar development, and cancer-associated stemness in various cancer types, including ATL (Sparmann and van Lohuizen, 2006).

The involvement of the Polycomb family in ATL was first revealed by global gene expression analysis. Significantly higher levels of enhancer of zeste homolog 2 (EZH2) as well as RING1 and YY1 binding protein (RYBP) transcripts with enhanced H3K27me3 levels were found in ATL cells compared with normal CD4+ T cells (Sasaki et al., 2011). EZH2 serves as the catalytic subunit in the PRC2 and mediates gene silencing by catalyzing the trimethylation of H3K27 at the promoters of target genes. EZH2 is highly expressed in many cancer types, including breast and prostate cancer and lymphomas, and it is often correlated with advanced stages of tumor progression and a poor prognosis. Importantly, EZH2 inhibition by 3-deazaneplanocin A and the HDAC inhibitor panobinostat showed a synergistic effect in killing the ATL cell lines. Because the Polycomb family generally contributes to silencing of tumor suppressor genes, e.g., the *CDKN2* family, the genes silenced in ATL should be addressed to elucidate the functional significance of the Polycomb family in the leukemogenic process.

We recently identified a notable gene silenced by Polycomb. A human gene that encodes miR-31, *hsa-miR-31*, is located at 9p21.3, which is adjacent to clusters of the *CDKN2* and *IFNA* families. In addition to the genetic loss (12.5% of ATL cases), transcription of the miR-31 precursor is completely lost in ATL cells. Computational predictions and experimental evidence clearly demonstrated that an assembly of YY1 binding motifs upstream of the miR-31 region is responsible for the occupancy of the Polycomb family at the target region, which leads to H3K27me3-dependent transcriptional repression. Overexpression of EZH2 and suppressor of zeste 12 (SUZ12) homolog, components of PRC2, in ATL cells can induce and maintain the epigenetic silencing of miR-31. Of note, given that miR-31 is a master regulator of the ATL-specific gene expression pattern described above, Polycomb-mediated loss can influence gene expression downstream of miR-31 (Figure 4). Indeed, the amount of EZH2 and SUZ12 directly strengthens cellular miR-31 depletion, which in turn activates the NF-κB pathway through NIK induction and confers anti-apoptotic features to T cell (Yamagishi et al., 2012). It is noteworthy that the molecular and biological interconnections between Polycomb–miR-31–NF-κB are conserved in breast cancer cells and B lymphocytes. By organizing the new principle, various cell types may realize the more complex gene regulatory network required for maintenance and execution of cellular functions. Imbalance of this network probably switches the cell fate from one to another.

The origin of epigenetic reprogramming observed in ATL cells remains elusive. In addition to self-dysfunction of the epigenetic machinery, a possible mechanism is viral hijacking; HTLV-1 Tax can physically associate with the key histone modifiers HDAC1 (Ego et al., 2002), SUV39H1 (Kamoi et al., 2006), and SMYD3 (Yamamoto et al., 2011). However, at present, the possible influence of the Tax-epigenetic association on gene regulation is unknown. Governing the epigenetic system by Tax may disrupt gene expression, leading to chronic proliferation and abnormal survival of HTLV-1-infected cells. In the context of viral gene regulation, epigenetic changes, mainly DNA methylation, in the HTLV-1 provirus may facilitate ATL cell evasion of the host immune system by suppressing viral gene transcription (Koiwa et al., 2002; Taniguchi et al., 2005). Recent studies using *in vivo* models strongly suggested that Tax and also other viral proteins are directly linked to leukemogenesis, despite viral gene expression being rare in circulating leukemic cells in patients (Hasegawa et al., 2006; Ohsugi et al., 2007b; Banerjee et al., 2010; Satou et al., 2011). Furthermore, not only histone methylation but also other histone modifications such as phosphorylation and ubiquitination are intriguing for understanding the molecular and physiological hallmarks of ATL.

## Therapeutic Targeting of ATL

**Figure 5:**
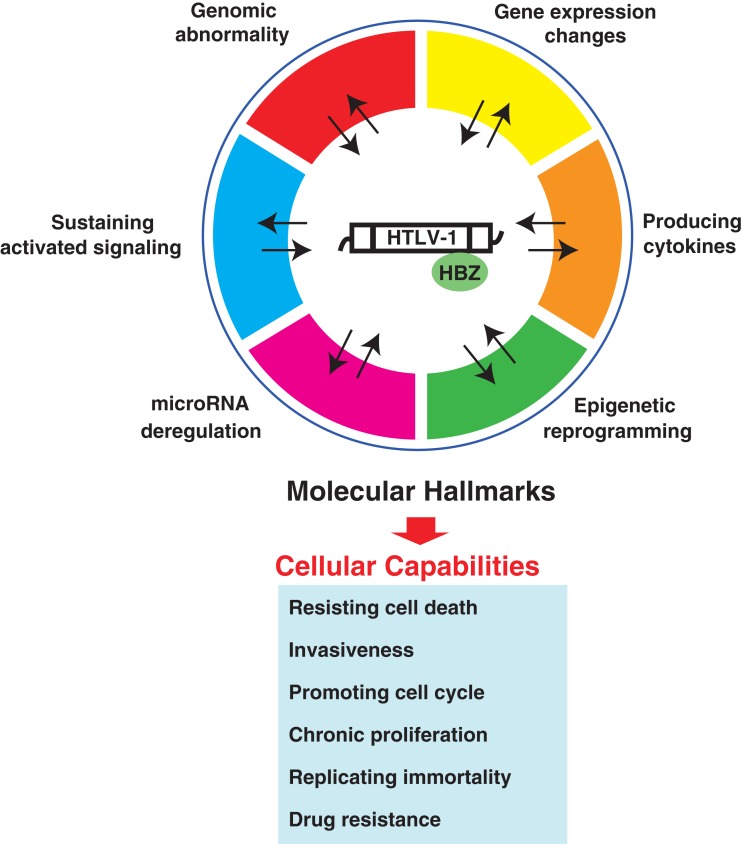
**Conceptual illustration of the molecular hallmarks of ATL**. This illustration encompasses the six molecular hallmarks of ATL. These organized principles provide characteristics of ATL itself. Because they may be directly associated with the clinical traits of ATL, targeting the one outstanding hallmark or co-targeting of multiple molecular hallmarks in mechanism-guided combinations will result in more effective and durable therapies for aggressive ATL.

To establish more effective molecular-targeted therapies for ATL, we need to understand the exact molecular underpinnings of ATL. In addition to classical molecular characteristics, the emerging hallmarks of miRNA deregulation and epigenetic reprogramming broaden the scope of conceptualization of the responsible molecular mechanism (Figure 4). As highlighted in this review, ATL possesses six molecular hallmarks: genomic abnormality, specific changes in gene expression, sustaining activated signaling, producing cytokines, miRNA deregulation, and epigenetic reprogramming. These molecular hallmarks confer robustness to leukemic cell hallmark capabilities: resisting cell death, promoting cell cycle, invasiveness, chronic proliferation, replicating immortality, and drug resistance (Figure 5). Consideration of hallmark principles should aid in developing future therapeutics. Several studies with inhibitory agents have clearly indicated that blockade of signaling drivers appears to be both practical and feasible for inducing leukemic cell apoptosis. However, common clinical traits of ATL include relapse and drug resistance. Importantly, each of the core hallmark capabilities is regulated by a partially redundant signaling pathways. Consequently, a targeted therapeutic agent that inhibits only one key pathway in ATL may not completely shut off another hallmark capability, allowing some ATL cells to survive with residual function until they or their progeny eventually adapt to the selective pressure imposed by the therapy. In this case, given that the number of parallel signaling pathways supporting a given hallmark is limited, it may become possible to target all of these supporting pathways therapeutically, thereby preventing the development of adaptive resistance. However, it is possible that this could involve critical side effects. Alternatively, most upstream elements that can act pleiotropically in leukemic cells, e.g., miRNA and epigenetics, may be heralded as one of the fruits of remarkable progress into understanding the ATL mechanism. Moreover, selective co-targeting of multiple core and emerging molecular hallmarks in mechanism-guided combinations therapies will result in more effective and durable therapies for aggressive ATL.

## Conclusion and Future Direction

We have explored our present understanding of the molecular aspects of ATL to refine and extend the six specific traits, the molecular hallmarks of ATL, which have provided a useful conceptual framework for understanding the complex biology of ATL (Figure 5).

Other areas are currently in rapid flux. In recent years, the biological importance of several elaborate ATL models, including the Tax-transgenic model (Hasegawa et al., 2006), HBZ transgenic model (Satou et al., 2011), and HTLV-1-infected humanized SCID mice (Banerjee et al., 2010), has been proposed. ATL-initiating stem cell theory has also been developed (Yamazaki et al., 2009; El Hajj et al., 2010). Similar to other lymphomas and solid cancers, leukemic cells in tissues may be encompassed by a tumor microenvironment that contributes to leukemogenesis. The organized principles of the molecular basis of ATL may be helpful in the coming decade of ATL study.

## Conflict of Interest Statement

The authors declare that the research was conducted in the absence of any commercial or financial relationships that could be construed as a potential conflict of interest.
